# Quality of life associated with chronic cough in the multinational Burden of Obstructive Lung Disease study: a cross-sectional study

**DOI:** 10.1183/23120541.00453-2025

**Published:** 2025-12-01

**Authors:** Hazim Abozid, Emiel F.M. Wouters, Hamid Hacene Cherkaski, Rune Nielsen, Marie-Kathrin Breyer, Kevin Mortimer, Karima El Rhazi, Gregory Erhabor, Asaad A. Nafees, Parvaiz Koul, Rana Ahmed, David Mannino, Cristina Barbara, Fatima Rodrigues, Abdul Rashid, Mahesh Padukudru Anand, Sanjay Juvekar, Dhiraj Agarwal, Wan C. Tan, Frits M.E. Franssen, Meriam Denguezli, Imed Harrabi, Christer Janson, Stefanni Nonna M. Paraguas, Terence Seemungal, Andre F.S. Amaral

**Affiliations:** 1 Ludwig Boltzmann Institute for Lung Health, Vienna, Austria; 2 Department of Respiratory and Pulmonary Diseases, Site Penzing of Clinic Ottakring, Vienna Healthcare Group, Vienna, Austria; 3 NUTRIM, Maastricht University Medical Center, Maastricht, the Netherlands; 4 Sigmund Freud Private University, Faculty of Medicine, Vienna, Austria; 5 Department of Pulmonology, Faculty of Medicine, University Badji Mokhtar, Annaba, Algeria; 6 Department of Thoracic Medicine, Haukeland University Hospital, Bergen, Norway; 7 Department of Clinical Science, University of Bergen, Bergen, Norway; 8 University of Cambridge, Cambridge, UK; 9 Liverpool University Hospitals NHS Foundation Trust, Liverpool, UK; 10 Department of Epidemiology and Public Health, Faculty of Medicine, Dentistry and Pharmacy, Laboratory of Research in Epidemiology and Health Sciences, Sidi Mohamed Ben Abdillah University, Hassan II University Hospital Center of Fez, Fez, Morocco; 11 Department of Medicine, Obafemi Awolowo University/Obafemi Awolowo University Teaching Hospitals Complex, Ile-Ife, Nigeria; 12 Department of Community Health Sciences, The Aga Khan University, Karachi, Pakistan; 13 Department of Pulmonary Medicine, Sheri Kashmir Institute of Medical Sciences, Srinagar, India; 14 The Epidemiological Laboratory, Khartoum, Sudan; 15 Royal College of Surgeons, School of Population Health, Dublin, Ireland; 16 University of Kentucky, Lexington, KY, USA; 17 COPD Foundation, Miami, FL, USA; 18 Instituto de Saúde Ambiental, Faculdade de Medicina, Universidade de Lisboa, Lisbon, Portugal; 19 Serviço de Pneumologia, Centro Hospitalar Universitário Lisboa Norte, Lisbon, Portugal; 20 Institute of Environmental Health, Associate Laboratory TERRA, Lisbon Medical School, Lisbon University, Lisbon, Portugal; 21 Royal College of Surgeons in Ireland and University College Dublin Malaysia Campus, Penang, Malaysia; 22 Department of Respiratory Medicine, JSS Medical College, JSSAHER, Mysuru, India; 23 Vadu Rural Health Program, KEM Hospital Research Centre, Pune, India; 24 Dr D.Y. Patil Medical College, Hospital and Research Centre, Dr D.Y. Patil Vidyapeeth, Pimpri, India; 25 Department of Medicine, Centre for Heart, Lung Innovation University of British Columbia, Vancouver, BC, Canada; 26 Department of Respiratory Medicine, Maastricht University Medical Center, Maastricht, the Netherlands; 27 Université de Monastir, Faculté de Médecine Dentaire de Monastir, Monastir, Tunisa; 28 Ibn El Jazzar Faculty of Medicine of Sousse, University of Sousse, Sousse, Tunisia; 29 Department of Medical Sciences, Respiratory Allergy and Sleep Research, Uppsala University, Uppsala, Sweden; 30 Philippine College of Chest Physicians, Manila, Philippines; 31 Philippine Heart Centre, Manila, Philippines; 32 Department of Clinical Medical Sciences, The University of The West Indies, St Augustine, Trinidad and Tobago; 33 National Heart and Lung Institute, Imperial College London, London, UK; 34 NIHR Imperial Biomedical Research Centre, London, UK

## Abstract

**Background:**

Chronic cough (CC) can impact daily life and persist for years. Its prevalence varies globally, but whether quality of life in CC also varies across regions is unknown. This study investigates the association of CC with mental and physical component scores of the 12-item Short Form Health Survey reflecting health-related quality of life in a multinational study.

**Methods:**

We analysed data from 19 642 adults (≥40 years), recruited between 2 January 2003 and 26 December 2016 in 31 sites (25 countries) from the Burden of Obstructive Lung Disease study, who provided information on quality of life and CC. We assessed associations using linear regression, adjusted for confounders, and used random-effects meta-analysis to examine differences by sex and gross national income.

**Findings:**

Overall, lower mental (−1.42, 95% CI −2.11 to −0.73; I^2^=32.7%) and physical (−2.59, 95% CI −3.22 to −1.96; I^2^=40.1%) health scores were associated with CC. The association between physical health score and CC did not materially differ between sexes or gross national income. In males, physical health seems to be more affected by CC amongst those living in low- and middle-income countries (LMICs). In females, mental health also seems to be more affected by CC amongst those living in LMICs.

**Interpretation:**

CC impairs health-related quality of life globally. However, it appears that physical health in males and mental health in females living in LMICs may be particularly affected by CC. These findings support the need to consider CC as a target for specific interventions to attenuate its burden on health and the economy.

## Shareable abstract

**These findings indicate the need for increased awareness about chronic cough and its association with reduced quality of life, particularly in low- and middle-income countries** https://bit.ly/4djqAhM

## Introduction

Chronic cough (CC) is a debilitating condition that impacts daily life and has been associated with poor quality of life (QoL) outcomes in some populations [1–3]. It has been associated with several other conditions including depression, anxiety, sleep disorders, urinary incontinence and social disability [4–9]. CC is also linked with high healthcare utilisation and costs [10–13]. In a European Lung Foundation internet survey conducted between 2012 and 2013, 96% of 1066 respondents said that their cough has an impact on their QoL [14]. Recent data from the population-based Respiratory Health in Northern Europe (RHINE) cohort show that participants with CC have lower work ability and are more likely to take more days of sick leave compared to those without CC [15].

Most reports on QoL associated with CC are from countries in Europe [12, 16–23] and North America [13, 24–28], as well as from China, Japan and South Korea [2, 11, 29–34]. With the exception of China, these are all high-income countries. In addition, different instruments to assess QoL, including generic scores as the 36-item Short Form Health Survey (SF-36) questionnaire [34, 35] and cough-specific tools like the Leicester Cough Questionnaire (LCQ), have been used [19, 22]. Therefore, the aim of this study was to investigate the association of CC with QoL across several world regions, including low- and middle-income countries (LMICs), using data from the multinational Burden of Obstructive Lung Disease (BOLD) study using the same tool across study sites [36].

## Methods

### Study design

A detailed description of the BOLD study has been published elsewhere [36]. In brief, non-institutionalised adults (≥40 years old) were identified and recruited between 2003 and 2016 from the general population in 41 sites with >150 000 inhabitants. In each site, the aim was to recruit a minimum of 600 participants, with an equal number of males and females. Information on respiratory symptoms, health status and exposure to potential risk factors was collected by trained fieldworkers, who administered standardised questionnaires translated into the local language. All sites received approval from their local ethics committee, and participants provided written informed consent. The study was conducted as per good clinical practice as well as local ethics regulations.

### Chronic cough

CC was defined as a cough, without having a cold, on most days for at least 3 months each year. It was assessed using two questions: “Do you usually cough when you don't have a cold?” and “Do you cough on most days for as much as 3 months each year?”, and participants who affirmatively responded to both were classified as reporting CC [37].

### Quality of life

QoL was measured using the 12-item Short Form Health Survey (SF-12) in 31 of the 41 study sites. The SF-12 form includes questions on 12 items covering eight health-related QoL domains. Answers to the SF-12 form are scored, and the results are reported in terms of a physical component score and a mental component score, which range from 0 to 100, with 100 representing the highest level of QoL [38].

### Statistical analysis

We used multivariable linear regression models to assess the association of the SF-12 component scores with CC in each study site. These models were adjusted for age (years), sex (male, female), smoking status (never, former, current), passive smoking (yes, no), body mass index (BMI) (underweight: <18.5 kg·m^−2^, normal weight: 18.5 to 24.9 kg·m^−2^, overweight: 25.0 to 29.9 kg·m^−2^, obese: ≥30.0 kg·m^−2^), exposure to dust in the workplace (years), education (years), a history of tuberculosis (yes, no), hypertension (yes, no), chronic airflow obstruction (post-bronchodilator ratio of the forced expiratory volume in 1 s to the forced vital capacity below the lower limit of normal (FEV_1_/FVC<LLN)), diabetes (yes, no), heart disease (yes, no) and dyspnoea (grade 2 or above on the modified Medical Research Council dyspnoea scale [39]). Site-specific estimates were pooled using random effects meta-analysis [40], and stratified by sex and smoking status. We used the I^2^ statistics to summarise heterogeneity across sites. Results were considered significant if p-value was <0.05. Analyses were conducted using Stata v.17 (Stata Corp., College Station, TX, USA).

## Results

### Population characteristics

Data on 19 642 participants who completed the core questionnaire and had data on CC and QoL were included in this study (tables 1 and 2). The mean age of participants ranged from 47 years in Mysore (India) to 59 years in Tartu (Estonia) and Salzburg (Austria). The lowest proportion of male participants was found in Tartu (Estonia) at 40.5% while the highest proportion was in Mumbai (India) at 62.9%. Mean BMI ranged from 21.6 kg·m^−2^ in Nampicuan-Talugtug (Philippines) to 30.9 kg·m^−2^ in Riyadh (Saudi Arabia). The prevalence of ever-smokers ranged from 9.6% in Mysore (India) to 69.6% in Uitsig-Ravensmead (South Africa).

**TABLE 1:** Characteristics of study participants in the Burden of Obstructive Lung Disease study

	n	Males %	Age years, mean±sd	Body mass index kg·m^−2^, mean±sd	Ever-smokers %	Passive smoking %	Education years, mean±sd	Dust in workplace years, mean±sd
**Albania (Tirana)**	887	49.5	55±12	27.5±4.1	36.5	37	10±5	15±14
**Algeria (Annaba)**	828	51.0	53±11	28.1±5.6	39.7	10.5	8±6	6±10
**Australia (Sydney)**	474	46.0	58±13	27.7±5	52.3	11.1	11±3	4±9
**Austria (Salzburg)**	1202	46.8	59±12	26.2±4.1	53.3	21.6	10±2	5±12
**Canada (Vancouver)**	793	47.9	56±12	26.7±5	57.3	5.6	16±3	3±8
**England (London)**	642	45.6	57±12	27.1±4.9	65.5	17.3	14±4	4±10
**Estonia (Tartu)**	565	40.5	59±12	28.3±5.3	45.6	15.3	14±4	5±10
**Germany (Hannover)**	529	47.0	57±11	26.6±4.3	60.4	17.8	11±2	3±9
**Iceland (Reykjavik)**	726	52.0	57±12	27.8±4.9	66.3	16.7	13±4	4±10
**India (Kashmir)**	751	51.6	51±11	22.6±3.7	52.2	64.1	2±4	0±1
**India (Mumbai)**	434	62.9	53±10	23.9±4.1	10.0	0.9	9±5	1±6
**India (Mysore)**	592	42.3	47±7	24.7±3.8	9.6	0	9±6	1±4
**India (Pune Rural)**	828	60.0	52±10	22.1±3.9	12.5	11	4±4	2±6
**Malawi (Blantyre)**	359	54.4	50±9	24.4±5	16.2	3.3	9±4	3±7
**Malawi (Chikwawa)**	381	56.9	51±10	21.8±3.8	29.2	2.7	4±4	3±7
**Morocco (Fes)**	474	60.3	53±12	27.2±4.9	35.3	8.1	5±6	9±13
**The Netherlands (Maastricht)**	572	47.4	58±12	27.4±4.5	64.3	18.2	15±5	3±9
**Nigeria (Ife)**	841	55.1	53±11	25±5.1	14.6	1.7	10±6	5±10
**Norway (Bergen)**	574	49.0	57±12	26.4±4.2	64.6	22	13±3	7±12
**Philippines (Manila)**	825	46.5	52±11	24.5±4.7	56.8	49.4	10±4	7±11
**Philippines (Nampicuan and Talugtug)**	715	49.3	54±11	21.6±4.1	55.2	47.2	8±4	6±12
**Poland (Krakow)**	477	49.9	55±11	27.6±4.7	61.7	40.8	11±3	10±13
**Portugal (Lisbon)**	563	47.8	57±12	27.7±4.5	44.9	19.8	9±5	10±14
**Saudi Arabia (Riyadh)**	609	54.9	50±8	30.9±5.7	27.0	5.4	10±5	3±8
**South Africa (Uitsig/Ravensmead)**	763	44.2	53±10	27.5±7.3	69.6	49.9	8±3	7±10
**Sudan (Gezeira)**	372	57.2	52±10	26.9±17.6	27.7	11.1	6±5	5±11
**Sudan (Khartoum)**	479	53.1	53±11	26.3±6.3	21.7	7.5	7±6	3±8
**Sweden (Uppsala)**	488	47.5	58±11	26.7±4.3	59.0	5.8	13±4	5±11
**Trinidad and Tobago**	1051	50.0	55±11	28.7±9.3	29.8	22	11±4	7±11
**Tunisia (Sousse)**	433	60.7	51±9	27.8±5.2	52.8	30.4	9±5	11±13
**USA (Lexington, KY)**	415	46.3	56±12	30.4±6.3	61.0	26.6	13±3	8±12

**TABLE 2:** Prevalence of chronic cough and other conditions in the Burden of Obstructive Lung Disease study

	n	Chronic cough %	Dyspnoea %	Chronic airflow obstruction %	History of tuberculosis %	Heart disease %	Hypertension %	Diabetes %
**Albania (Tirana)**	887	9.8	9.2	7.4	0.7	2.7	22.1	6.9
**Algeria (Annaba)**	828	2.8	12.3	6.2	2.4	6.1	22.2	14.2
**Australia (Sydney)**	474	6.4	7.2	9.7	0.8	11.1	31.4	8.2
**Austria (Salzburg)**	1202	5.4	8	16.9	2.6	12.7	29.9	5.9
**Canada (Vancouver)**	793	10.6	6.7	12.7	3.1	11.9	20.2	7.4
**England (London)**	642	14.3	14.2	17	1.8	6.2	33.7	6.3
**Estonia (Tartu)**	565	6.7	13.5	5.9	6.2	32.4	36.5	6.3
**Germany (Hannover)**	529	7.7	3.8	8.2	3.6	12.7	33.2	4.8
**Iceland (Reykjavik)**	726	11.1	8.7	11.1	4.9	13.7	31.7	4.2
**India (Kashmir)**	751	5.6	4.9	16	0.4	1.1	26.7	2.6
**India (Mumbai)**	434	1.6	9.8	6.5	0.5	2	10.6	5.2
**India (Mysore)**	592	1.4	0.0	7.9	0	0.2	17	16.9
**India (Pune Rural)**	828	1.7	6.6	5.9	0.8	0.8	5.2	2.2
**Malawi (Blantyre)**	359	2.1	1.9	6.4	5.2	1.9	16.1	5.9
**Malawi (Chikwawa)**	381	1.2	1.4	12.2	4	0.9	1.6	1.3
**Morocco (Fes)**	474	6.4	14.8	6.8	0.9	2.9	22	9.1
**The Netherlands (Maastricht)**	572	5.3	10.3	18.4	1.5	16.4	30	7.2
**Nigeria (Ife)**	841	0.4	3.2	7	0.4	0.1	1.4	0.6
**Norway (Bergen)**	574	7.2	5.3	11.1	0.1	8.7	25.2	4
**Philippines (Manila)**	825	6.1	22.9	8.6	10.3	7.6	23.6	6.1
**Philippines (Nampicuan and Talugtug)**	715	8	28.8	15	3.5	7.4	20.4	2.6
**Poland (Krakow)**	477	7	24.1	13.9	2	29.8	40.2	10
**Portugal (Lisbon)**	563	9.1	12.2	7.8	5	9.3	28.9	8.9
**Saudi Arabia (Riyadh)**	609	10.9	22.5	2.8	1.8	5	24	27.4
**South Africa (Uitsig/Ravensmead)**	763	11.4	28.7	18.8	15.2	7.3	34.9	11.4
**Sudan (Gezeira)**	372	1.3	8.2	5.8	0.3	0	9.7	6.7
**Sudan (Khartoum)**	479	3.3	6.8	10.1	0.9	1.9	20.6	8.7
**Sweden (Uppsala)**	488	6.7	5.5	7.8	1.1	9.6	26.6	3
**Trinidad and Tobago**	1051	7.1	8.6	5.9	0	5.3	27.9	14.8
**Tunisia (Sousse)**	433	8.2	15.4	5.2	0	4	14.1	7
**USA (Lexington, KY)**	415	18.1	19.5	12.4	2.3	22.8	43.8	15.7

The prevalence of CC (table 2) varied widely, ranging from 0.4% in Ife (Nigeria) to 18.1% in Lexington, KY (USA). For other respiratory conditions, dyspnoea was most frequently reported in Nampicuan-Talugtug (Philippines) at 28.8%, while chronic airflow obstruction was most common in Uitsig-Ravensmead (South Africa) at 18.8%. Regarding cardiometabolic comorbidities, heart disease had the highest prevalence in Tartu (Estonia) at 32.4%, diabetes was most prevalent in Riyadh (Saudi Arabia) at 27.4%, and arterial hypertension was most common in Lexington (KY, USA) at 43.8%.

Table 3 provides the mean QoL component scores for each study site. For physical component scores, Krakow (Poland) had the lowest (43.9) while Blantyre (Malawi) had the highest (53.9) mean score. In terms of mental health, the lowest mean score (41.8) was found in Fes (Morocco), whereas the highest mean score (58.4) was found in Mysore (India).

**TABLE 3:** 12-item Short Form Health Survey (SF-12) mental and physical component scores in the Burden of Obstructive Lung Disease study

	n	Mental component score, mean±sd	Physical component score, mean±sd
**Albania (Tirana)**	887	51.4±5.9	51.5±6.8
**Algeria (Annaba)**	828	49.3±7.2	49.7±8.8
**Australia (Sydney)**	474	51.8±9.3	51.1±8.5
**Austria (Salzburg)**	1202	54.2±8.6	50.4±7.5
**Canada (Vancouver)**	793	50.8±9.6	51.6±9
**England (London)**	642	48.8±11	49±10.3
**Estonia (Tartu)**	565	52±8.7	47.3±9.2
**Germany (Hannover)**	529	55.9±7.5	48.6±7.9
**Iceland (Reykjavik)**	726	53.6±8.9	50.5±9.3
**India (Kashmir)**	751	51.7±6.3	51.2±6.6
**India (Mumbai)**	434	58±6.6	52.3±7.1
**India (Mysore)**	592	58.4±7.1	53.3±4.1
**India (Pune Rural)**	828	49.3±7.4	50.1±6.6
**Malawi (Blantyre)**	359	54±8.2	53.9±3.9
**Malawi (Chikwawa)**	381	56±7.9	52.3±4.1
**Morocco (Fes)**	474	41.8±8.4	50.8±9.2
**The Netherlands (Maastricht)**	572	53.5±9.4	50.2±9
**Nigeria (Ife)**	841	55.2±9.7	45.5±8.3
**Norway (Bergen)**	574	54.2±8.9	50.8±8.7
**Philippines (Manila)**	825	53.2±9.4	46.4±7.6
**Philippines (Nampicuan and Talugtug)**	715	50.1±7.2	45.7±7.6
**Poland (Krakow)**	477	47.8±10.2	43.9±10.7
**Portugal (Lisbon)**	563	50.3±11.4	49.7±8.6
**Saudi Arabia (Riyadh)**	609	50.2±7.8	48.5±8.6
**South Africa (Uitsig/Ravensmead)**	763	49±9.8	46.5±9.4
**Sudan (Gezeira)**	372	53.1±10.1	47.3±7.6
**Sudan (Khartoum)**	479	47.6±9.2	48.8±7.4
**Sweden (Uppsala)**	488	43.5±6.8	48.8±6.6
**Trinidad and Tobago**	1051	54.9±8.4	48.2±7.1
**Tunisia (Sousse)**	433	50.5±9.7	47.4±8.6
**USA (Lexington, KY)**	415	50.4±11.1	45.8±11.4

### Physical health and chronic cough

**FIGURE 1:**
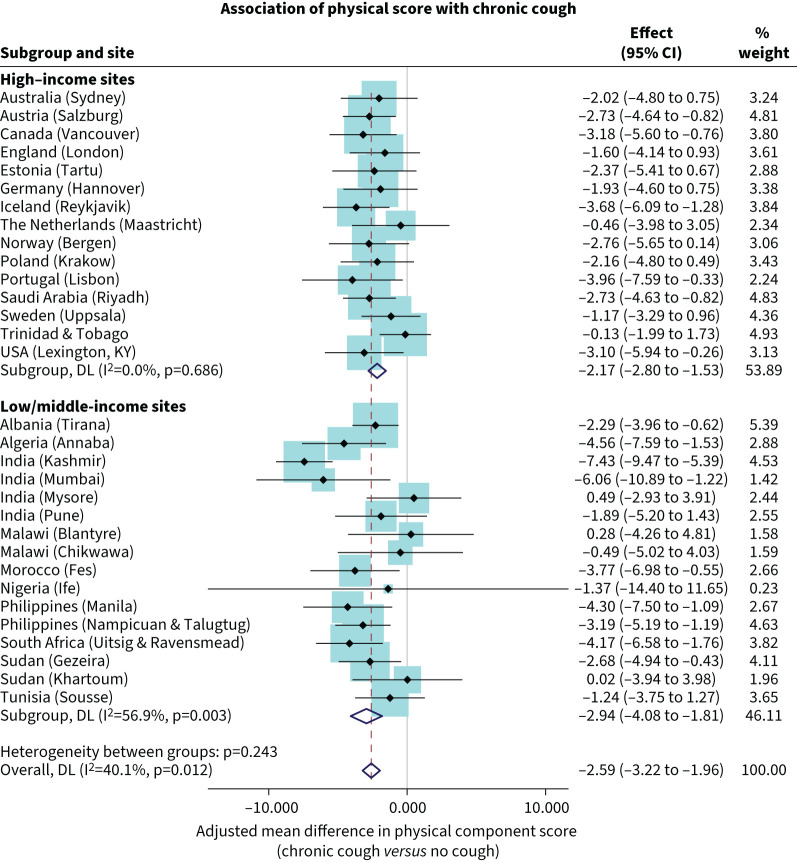
Association of physical health score with chronic cough in the Burden of Obstructive Lung Disease (BOLD) participants. Effect size (95% CI) is the adjusted odds ratio and 95% confidence interval. DL: “DerSimonian–Laird” random-effects model.

The physical component score in people with CC was lower than that of people without CC (−2.59, 95% CI −3.22 to −1.96) (figure 1). The heterogeneity across sites was low (I^2^=40.1%) and mainly driven by stronger associations in Kashmir (India) with a difference of −7.43 (95% CI −9.47 to −5.39) and Mumbai (India) with −6.06 (5% CI −10.89 to −1.22). There was no material difference in the association of physical health with CC between high-income country sites (−2.17, 95% CI −2.80 to −1.53) and LMIC sites (−2.94, 95% CI −4.08 to −1.81). The magnitude of the association between physical component score and CC was approximately the same in females (−2.44, 95% CI −3.27 to −1.61) (supplementary figure S1) and in males (−2.93, 95% CI −4.02 to −1.83) (supplementary figure S2). Although not statistically significant, physical scores in association with CC were lower among males from LMIC sites than in high income sites.

### Mental health and chronic cough

**FIGURE 2:**
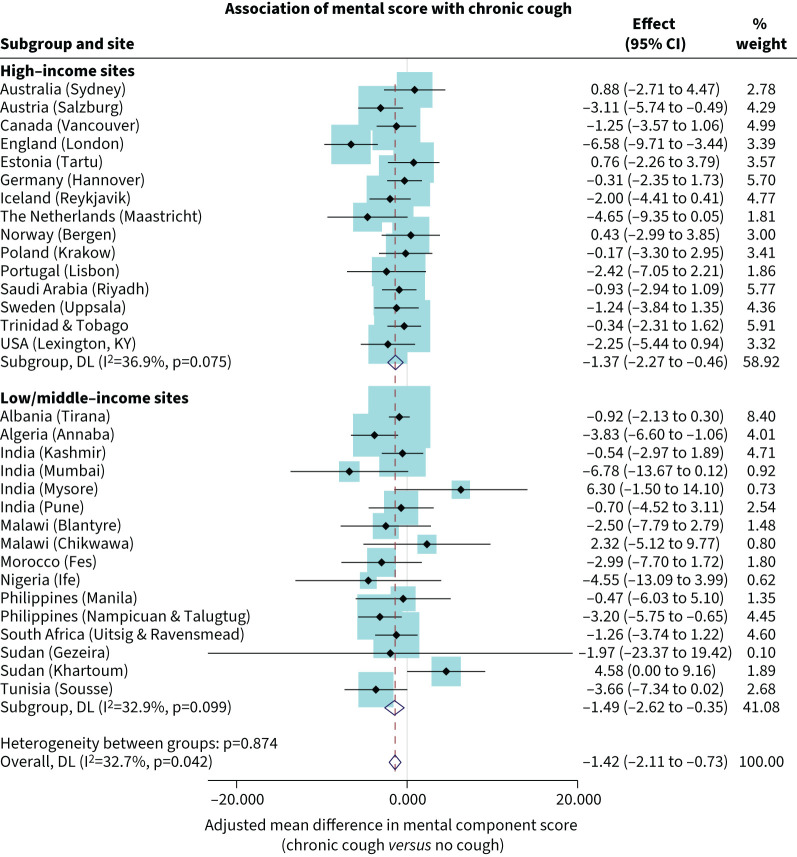
Association of mental health score with chronic cough in the Burden of Obstructive Lung Disease (BOLD) participants. Effect size (95% CI) is the adjusted odds ratio and 95% confidence interval. DL: “DerSimonian–Laird” random-effects model.

The mental component score in people with CC was lower than that of people without CC (−1.42, 95% CI −2.11 to −0.73) (figure 2). The heterogeneity across sites was low (I^2^=32.7%) and mainly driven by Mumbai (India) with a difference of −6.78 (95% CI −13.67 to 0.12), London (England) with −6.58 (95% CI −9.71 to −3.44) and Maastricht (the Netherlands) with −4.65 (95% CI −9.35 to 0.05), as well as Mysore (India) with 6.30 (95% CI −1.50 to 14.10) and Khartoum (Sudan) with 4.58 (95% CI 0.00 to 9.16). There was no difference in the association of mental health with CC between high-income country sites (−1.37, 95% CI −2.27 to −0.46) and LMIC sites (−1.49, 95% CI −2.62 to −0.35).

The magnitude of the association between mental component score and CC was approximately the same in females (−1.19, 95% CI −2.22 to −0.16) (supplementary figure S3) and in males (−1.71, 95% CI −2.79 to −0.64) (supplementary figure S4). Although not statistically significant, mental health scores were lower among females from LMIC sites than in high income sites.

## Discussion

In this multinational population-based study, CC was associated with worse physical and mental health-related QoL. These associations were independent of chronic airflow obstruction, dyspnoea, history of tuberculosis and cardiometabolic comorbidities.

We report lower physical and mental health scores in individuals with CC compared to those without. One of the key findings was that CC has a particularly greater impact on the physical component, which can also be observed in other respiratory conditions [41–45]. For CC, similar findings of studies that also used SF-12, lower physical component scores have been reported from China [2], Germany [21] and Norway [46], while data reporting a stronger association with the SF-12 mental component score are from Japan [11], South Korea and Taiwan [47], the UK [12], France [20] and Spain [23]. The use of other cough-specific tools like the LCQ also reflects this variability: a study from the Netherlands [22] reported lower physical domain scores, while a Finnish employee study [19] found slightly lower psychological scores. Using the SF-36 questionnaire also showed different directions where lower physical scores were reported in Australia [35] and Korea [48], but lower mental health scores in China [34].

The association with physical and mental QoL was significant and relatively of the same magnitude in both sexes. This contradicts prior studies suggesting that females may experience a greater impact of CC on health-related QoL [30] because they have a heightened cough reflex sensitivity [49, 50] and are more likely to seek medical attention due to other physical complaints like urinary stress incontinence [22, 51, 52]. Our results are supported by findings from primarily Asian studies stating that females did not have a greater impact on physical health scores [2, 34] despite reporting more frustration and sleep disturbance compared to men [34]. It is also reported that females are more adversely affected as the duration of a cough increases [53]. Considering this, previous studies that reported a stronger negative impact on females may have experienced potential selection bias by including females with a higher frequency of coughing [34]. However, slight sex differences without statistical significance were observed in LMIC sites, where lower physical QoL scores were reported in males and lower mental QoL scores in females.

To our knowledge, no study has compared the effect of CC on QoL by national income levels. Our study provides strong evidence that CC is a significant global health issue with a substantial negative impact on both physical and mental QoL without a clear pattern indicating that CC has a stronger impact in either high-income sites or LMIC sites. However, the above mentioned sex-related differences were more pronounced in LMICs where healthcare resources are commonly more limited and chronic respiratory symptoms are among the most prevalent and increasing health complaints [54–56].

The strengths of this population-based study include its large sample size and wide geographical coverage, the use of a standardised protocol for data collection across study sites, and the rigorous training of all staff involved in the study. Our study also has limitations. Our definition of CC (*i.e.* 3-month cut-off for cough duration) is not in line with the most recent guidelines [5, 57–59], but it agrees with the majority of epidemiological studies on CC [60]. This difference should be considered when comparing our findings with studies with more recent data collection. The cross-sectional design of this study limits the ability to establish causality. However, it is unlikely that low health-related QoL would cause CC. The wide variation in prevalence of CC across our study sites is likely explained by varying exposure to risk factors [37, 61–63], and because information on CC was self-reported, it may also be influenced by culture, health literacy and different thresholds for reporting cough [64]. However, this is unlikely to have had much influence in our findings as there was little evidence of heterogeneity in the association between QoL and CC across sites. CC can present with comorbidities such as gastroesophageal reflux, rhinosinusitis and depression [8, 17, 18, 65], for which we do not have information. We did, however, adjust our models for chronic airflow obstruction, which is a key characteristic of COPD. These conditions should be considered as potential confounders when assessing the relationship of CC with QoL in future studies. In addition, this study is limited to adults aged 40 years and above.

In conclusion, our study found that CC significantly impacts both physical and mental health-related QoL with a stronger effect on the physical component across world regions independently of other respiratory or cardiometabolic conditions. It also suggests that the strength of these associations may differ by sex depending on the gross national income of the country of residence. Our findings emphasise the need for further research into these associations and their underlying mechanisms so that interventions can be tailored accordingly and supports further research into the treatment of cough.

## Data Availability

De-identified participant data and questionnaires may be shared, after publication, on a collaborative basis upon reasonable request made to A.F.S. Amaral (a.amaral@imperial.ac.uk). Requesting researchers will be required to submit an analysis plan.

## Supplementary material

**Please note:** supplementary material is not edited by the Editorial Office, and is uploaded as it has been supplied by the author. Figure S1 00453-2025.SUPPLEMENT Figure S2 00453-2025.SUPPLEMENT2 Figure S3 00453-2025.SUPPLEMENT3 Figure S4 00453-2025.SUPPLEMENT4
