# Supplementary figures and images for: Quality of life associated with chronic cough in the multinational Burden of Obstructive Lung Disease study: a cross-sectional study
Source: ERJ Open Res. 2025 Dec 1;11(6):00453-2025. doi: 10.1183/23120541.00453-2025 (PMC12683565; doi:10.1183/23120541.00453-2025)

# Association of physical score with chronic cough among females

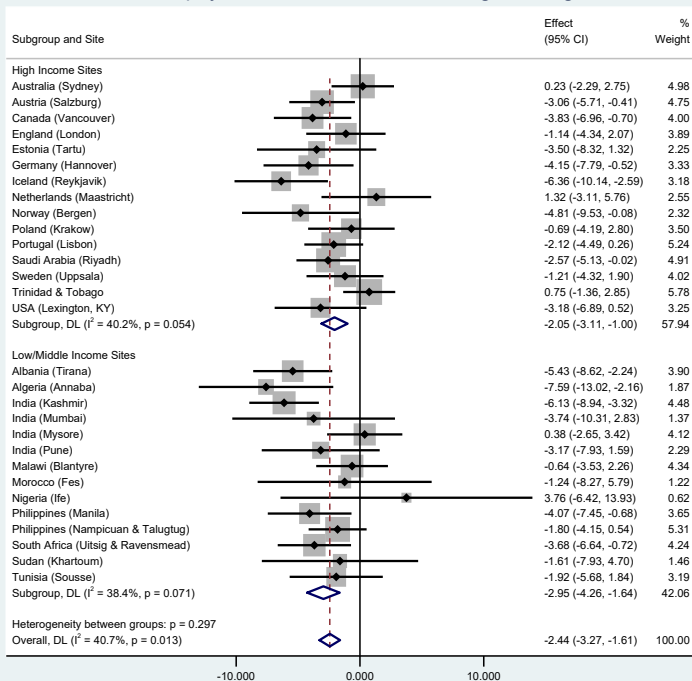

Supplement: Supplementary file 1 [file 00453-2025.SUPPLEMENT.pdf]

# Association of physical score with chronic cough among males

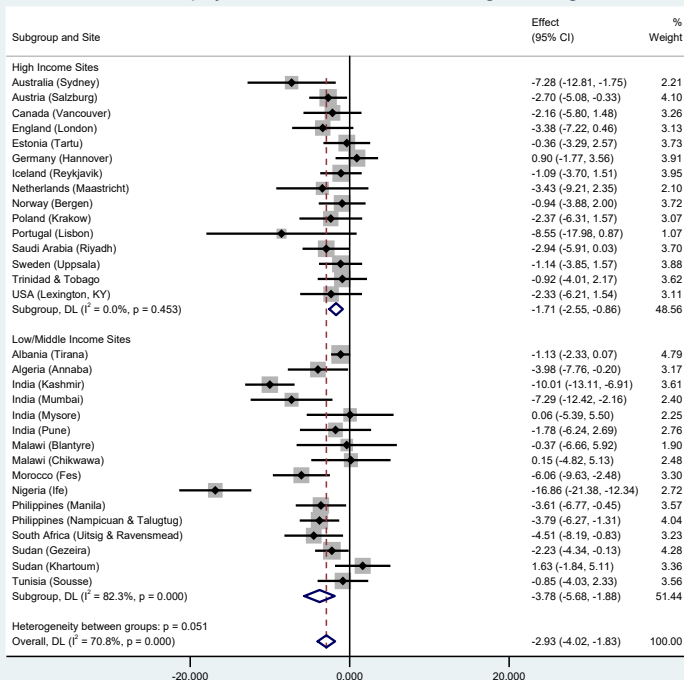

Supplement: Supplementary file 2 [file 00453-2025.SUPPLEMENT2.pdf]

# Association of mental score with chronic cough among females

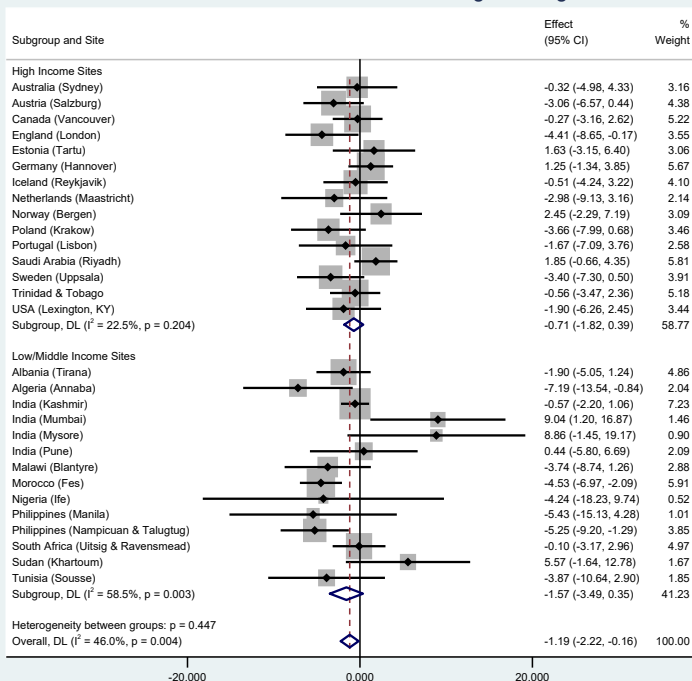

Supplement: Supplementary file 3 [file 00453-2025.SUPPLEMENT3.pdf]

# Association of mental score with chronic cough among males

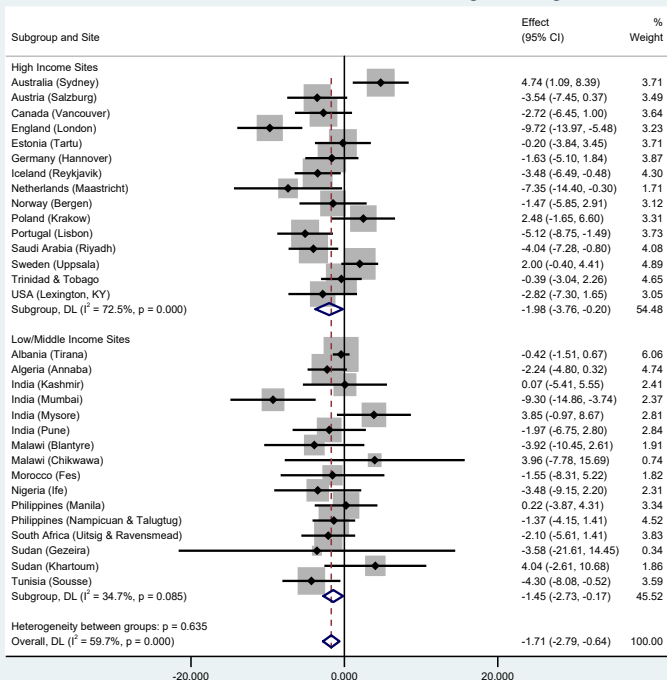

Supplement: Supplementary file 4 [file 00453-2025.SUPPLEMENT4.pdf]
